# Clinical Translation of Mesenchymal Stromal Cell Therapy for Graft Versus Host Disease

**DOI:** 10.3389/fcell.2019.00255

**Published:** 2019-11-21

**Authors:** Juliana A. P. Godoy, Raquel M. A. Paiva, Aline M. Souza, Andrea T. Kondo, Jose M. Kutner, Oswaldo K. Okamoto

**Affiliations:** ^1^Departamento de Hemoterapia e Terapia Celular, Hospital Israelita Albert Einstein, São Paulo, Brazil; ^2^Centro de Pesquisa sobre o Genoma Humano e Células-Tronco, Departamento de Genética e Biologia Evolutiva, Instituto de Biociências, Universidade de São Paulo, São Paulo, Brazil

**Keywords:** mesenchymal stromal cells, immunomodulation, graft versus host disease, bone marrow, good manufacturing practices

## Abstract

Graft versus host disease (GVHD) is a common condition in patients subjected to allogeneic hematopoietic stem cell transplantation (HSCT). The immune cells derived from the grafted stem cells attack recipient’s tissues, including those from the skin, liver, eyes, mouth, lungs, gastrointestinal tract, neuromuscular system, and genitourinary tract, may lead to severe morbidity and mortality. Acute GVHD can occur within few weeks after the allogeneic cells have engrafted in the recipient while chronic GVHD may occur any time after transplant, typically within months. Although treatable by systemic corticosteroid administration, effective responses are not achieved for a significant proportion of patients, a condition associated with poor prognosis. The use of multipotent mesenchymal stromal cells (MSCs) as an alternative to treat steroid-refractory GVHD had improved last decade, but the results are still controversial. Some studies have shown improvement in the life quality of patients after MSCs treatment, while others have found no significant benefits. In addition to variations in trial design, discrepancies in protocols for MSCs isolation, characterization, and *ex vivo* manipulation, account for inconsistent clinical results. In this review, we discuss the immunomodulatory properties supporting the therapeutic use of MSCs in GVHD and contextualize the main clinical findings of recent trials using these cells. Critical parameters for the clinical translation of MSCs, including consistent production of MSCs according to Good Manufacturing Practices (GMPs) and informative potency assays for product quality control (QC), are addressed.

## Introduction

Allogeneic hematopoietic stem cell transplant (HSCT) is a treatment for high risk hematological and malignant diseases. Conditioning regimen, immunosuppressive strategies, supportive care and prophylaxis for infectious disease are improving, reducing mortality related to transplant (Appelbaum, 2001). However, graft versus host disease (GVHD) remains one of the most common complication with high rate of disability and mortality (Perkey and Maillard, 2018).

**FIGURE 1:**
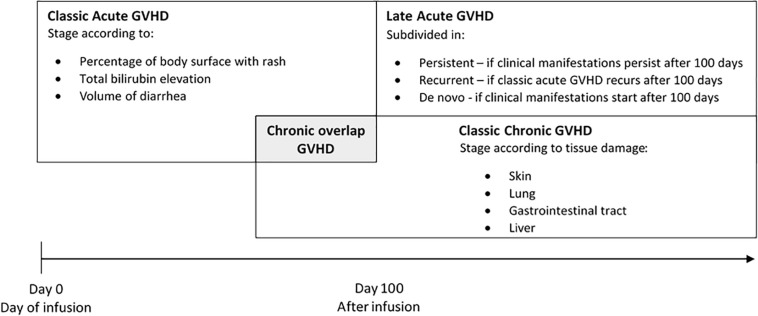
Classification and stage of graft versus host disease (GVHD).

Graft versus host disease occurs when immunologically competent donor T lymphocytes recognize recipient’s tissue as foreign resulting in damage in many organs systems including skin, liver, gastrointestinal tract, and lung. Clinical manifestations are classified as acute or chronic GVHD. In the past, all clinical manifestations of GVHD occurring before 100 days of transplant where classified as acute GVHD. When clinical manifestations occurred later on, after 100 days of transplant, it was considered chronic GVHD. In 2005, the National Institutes of Health (NIH) Consensus Conference determined new criteria of diagnosis and scoring and abolished the 100 days criterion. NIH Consensus considered acute and chronic GVHD as two distinguished symptoms without restriction of time (Filipovich et al., 2005; Figure 1).

Among all patients undergoing allogeneic HSCT 30–50% will have acute GVHD (grade 1–4) and 14% of patients exhibit chronic GVHD. The most important acute GVHD risk factor is human leukocyte antigen (HLA) mismatch (Petersdorf et al., 1995; Flomenberg et al., 2004). Transplants involving female donor/male recipient or unrelated donor are also associated with higher risk of developing late acute and chronic GVHD (Arora et al., 2016).

Pathophysiology of acute GVHD involves engraftment of immunocompetent cells in a host with mismatched antigens that is incapable to respond against graft cells, allowing donor lymphocytes activation to attack host tissue (Billingham, 1966). The damage to host tissues leads to production of proinflammatory cytokines, such as tumor necrosis factor (TNF) α, interleukin (IL) 1, 2, and 6, chemokines and increased expression of adhesion molecules, costimulatory molecules and major histocompatibility complex (MHC) antigens on the tissue (Jamil and Mineishi, 2015). Regulatory T Cells (Tregs) have been shown to downregulate the alloreactivity of T cells *in vitro* and *in vivo* and natural killer cells (NK cells) have been shown to modulate GVHD in a clinical trial reducing incidence of GVHD (Zeng et al., 1999; Cohen and Boyer, 2006).

The pathophysiology of chronic GVHD is more complex. All mechanisms reported in acute GVHD are relevant, however, other pathways are under investigation. The presence of auto and alloantibodies is described but is unclear whether these antibodies are involved in pathogenesis or are just markers of B cell dysregulation (Shimabukuro-Vornhagen et al., 2009). The presence of these auto antibodies is also described along with implication of Treg dysfunction in the development of chronic GVHD (Martin, 2008).

Acute and chronic GVHD are first treated by glucocorticoids. However, 50–60% of patients are resistant to glucocorticoids (Flowers and Martin, 2015; Mielcarek et al., 2015) and they have poor long-term prognosis with overall survival rate of only 5–30% (Zeiser and Blazar, 2017). Alternative treatments involve different immunosuppressive drugs like Calcineurin inhibitor, Antithymocyte globulin (ATG), Anti-interleukin 2 receptor antibodies, Anti-TNFα agents, Extracorporeal photopheresis (ECP), Mycophenolate mofetil (MMF), Sirolimus, and Pentostatin. None of them are fully effective and new therapeutic modalities for refractory GVHD are currently under investigation, including therapy with mesenchymal stromal cells (MSCs).

## Mesenchymal Stromal Cell Identity

After their first description in bone marrow by Friedenstein et al. (1968), mesenchymal cells were later found to reside in almost all post-natal tissues, being recruited to sites of tissue injury. Although at variable quantities, mesenchymal stem cells are also isolated from cord blood (Erices et al., 2000), umbilical cord (Wang et al., 2004), amnion (Kaviani et al., 2001), placenta (Fauza, 2004), peripheral blood (Kassis et al., 2006), adipose tissue (Zuk et al., 2002), dental pulp (Gronthos et al., 2000), maternal milk (Patki et al., 2010), skin (Shih et al., 2005), and menstrual blood (Meng et al., 2007), among others. However, the great variability in the protocols for mesenchymal stem cell isolation and *ex vivo* expansion may sometimes result in cultures of cells with distinct properties.

In attempt to help standardize the growing research field with such mesenchymal cells, the International Society for Cellular Therapy suggested using the term “MSCs,” due to the lack of uniform evidences for their stem cell activity (Horwitz et al., 2005). The same Society also proposed minimum criteria to characterize MSCs, namely culture plastic adherence, ability to differentiate *in vitro* into adipocytes, chondrocytes and osteocytes, and expression of specific membrane surface antigens (Dominici et al., 2006).

Although widely accepted, these criteria do not guarantee purity of MSC preparations since other cell types, such as fibroblasts, to some extent comply with these same requirements (Junker et al., 2010; Pereira et al., 2011). Heterogeneity in MSC products may lead to discrepant clinical outcomes. Indeed, in an experimental model of Parkinson’s Disease, contamination of MSC preparations with fibroblasts abolished MSC-induced therapeutic effects and enhanced degeneration of dopaminergic neurons (Pereira et al., 2011). Therefore, defining clear threshold levels of critical cell parameters may improve MSC quality testing. Assessment of alternative membrane markers enriched in MSCs compared to other cell types, such as CD166 (Halfon et al., 2011), CD271 (Jones et al., 2002), or CD146 (Sacchetti et al., 2007) have also been proposed for MSC immunophenotyping.

Therefore, following strict criteria for MSC identity is essential for comparability and reproducibility studies. Nonetheless, it is also important to continuously revise these consensus criteria once knowledge is updated in the literature.

## Immunomodulatory Properties of Mesenchymal Stromal Cells

Mesenchymal stromal cells are highly metabolically active, secreting not only extracellular matrix molecules (Wight et al., 1986), but also a variety of cytokines (Horwitz and Dominici, 2008). Indeed, the paracrine effects of MSCs, such as those related to regulation of immune response, seem more relevant under certain physiopathological conditions than their multipotency. Some studies reported that MSCs are able to affect the activity of both, T and B cells. MSCs may suppress T cell proliferation, cytokine release, cytotoxicity, and Th1/Th2 balance (Puissant et al., 2005; Selmani et al., 2008). MSCs also affect B cells viability, antibodies secretion and the co-stimulatory production of molecules released by B cells (Corcione et al., 2006). Some studies have also reported MSC to be capable of inhibiting antigen maturation, activation and presentation by dendritic cells (Ramasamy et al., 2007), as well as inhibiting interleukin-2 (IL-2) production by NK cells (Spaggiari et al., 2006).

It is known that the immunomodulatory effects of MSCs depend on cell activation by stimulatory molecules in the microenvironment. The main factors leading to MSCs activation are IFN-gamma, TNF-α, and IL-1β (Krampera, 2011). The release and ligation of IFN-gamma to receptors in MSCs are key factors inducing immunomodulatory properties not only in T cells, but also in B and NK cells, which are not responsive to IFN-gamma by itself (Duffy et al., 2011; Franquesa et al., 2012).

**FIGURE 2:**
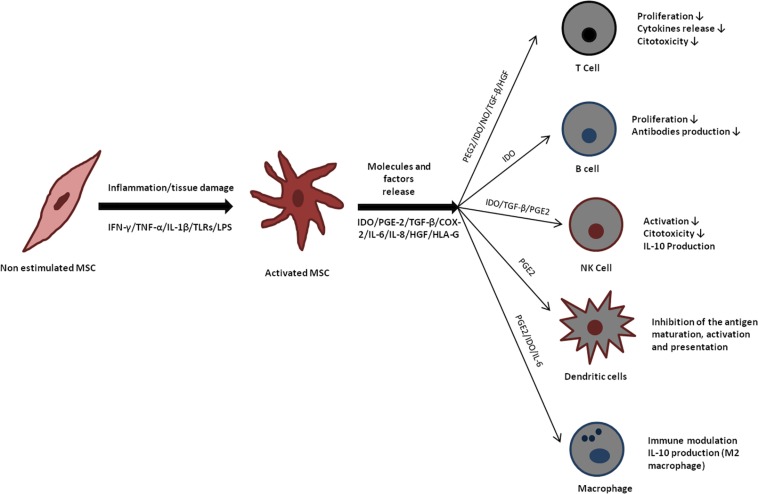
Immunomodulatory effects of mesenchymal stromal cells (MSC). Mesenchymal stromal cells release several molecules that act directly in cells from the immune system, favoring an anti-inflammatory microenvironment.

The stimulation by TNF-α or IL-1β cause significant modification in MSCs phenotype, which include MHC class I expression and increase in ICAM-1 and VCAM-1 expression (Ren et al., 2010). The combinatory action of IFN-gamma and TNF-α increases IL-6, IL-8, HGF, PGE-2, and cyclo-oxigenase-2 (COX-2) expression in MSCs (Hemeda et al., 2010). IFN-gamma action alone results in induction of MHC class II, indoleamine 2,3-dioxygenase (IDO) and PD-L1 expression (Sheng et al., 2008). Up-regulation of IDO has been shown to have therapeutic potential in preventing GvHD. IDO activity leads to production of kynurenine, a tryptophan breakdown product capable of inducing apoptosis of T cells and suppression of inflammation (Jasperson et al., 2009). Programed death 1 (PD-1) and its ligand (PD-L1) are important players in GVHD, by delivering inhibitory signals avoiding immune mediated tissue damage (Blazar et al., 2003). IFN-gamma and TNF-α co-activation induce expression of chemokines such as CCR5, CCR10, CXCR3, CXCL9, and CXCL10, which are involved in chemotaxis and may inhibit the proliferation of effector cells in the immune system (Ren et al., 2008; English, 2013; Najar et al., 2016). The interplay between pro-inflammatory factors, production/activation of immunomodulatory molecules by MSCs, and ensuing consequences on immune system cells is illustrated in Figure 2.

In a recent study using C57BL/6 mice, it was found that 24 h post-injection of umbilical cord MSCs in the tail vein, most cells were dead and located in lungs and liver, with a huge presence of Ly6C^low^ monocytes. *In vitro* assays showed that human monocytes were polarized from a CD14^++^/CD16^–^ to a CD14^++^/CD16^+^/CD206^+^ phenotype after MSCs phagocytosis. These monocytes also expressed PDL-1 and IL-10, while TNF-α was reduced. These modified monocytes after MSCs phagocytosis induced Treg Foxp3^+^ formation, indicating that monocytes play a key role in the MSCs immune modulatory response (de Witte et al., 2018). Some *in vitro* studies showed that MSCs stimulated monocytes to acquire an anti-inflammatory phenotype with IL-10 production (Melief et al., 2013; Deng et al., 2016). Gonçalves et al. (2017) demonstrated that particles derived from the plasma membrane of MSCs were able to induce pro-inflammatory monocytes to apoptosis, thereby modulating the immune response.

Apoptosis seems to be involved in different mechanisms leading to immunosuppression. Another study in a mouse model of GVHD reported that the immunosuppression effect of MSCs depends on the recipient’s cytotoxic T cell activity. It was found that highly cytotoxic T cells in the recipient induces MSC apoptosis and that apoptotic MSCs are cleared by recipient’s phagocytes. This process induces production of IDO by the phagocytes, thereby promoting immunosuppression (Galleu et al., 2017). In this study, the authors confirmed that the cytotoxic activity of GVHD patient’s T cells against MSCs was positively correlated with clinical response, which led them to propose the use of an *in vitro* cytotoxicity test against MSCs to help screening eligible GVHD patients to undergo treatment with MSCs.

As many signaling pathways involved in MSCs immunomodulatory effects are mediated by soluble factors, cell-free therapy strategies such as those with MSC-derived exosomes are also being considered. Exosomes can carry important active molecules such as cytokines, mRNAs and regulatory miRNAs (Yin et al., 2019). Exosomes released by bone marrow MSCs were reported to improve GVHD in mice by inhibiting CD4 T cells, reducing inflammatory cytokines and increasing IL-10 expressing Treg cells (Lai et al., 2018).

## Mesenchymal Stromal Cells for Graft Versus Host Disease

The better understanding of the MSCs immunomodulatory properties and of the GVHD pathophysiology has supported a rationale for potential benefits of a MSC-based therapy for this condition. Given that MSCs suppress proliferation of activated lymphocytes, reduce IFN-gamma production and upregulate activation markers (Klyushnenkova et al., 2005), in 2004, MSC was first used successfully to treat GVHD (Le Blanc et al., 2004). This first approach from a haploidentical third party donor showed that MSCs treatment could be safe and potentially effective. Since then, MSC has been studied to prove its efficacy, but the results are still controversial, probably as a consequence of variations in trial design.

## Source of Msc

Studies evaluating MSC for GVHD normally use allogeneic MSCs as patients usually don’t have clinical condition for this donation. In the beginning, HLA matched donors were selected, however, *in vitro* culture showed similar suppressive effects despite HLA matched (Le Blanc et al., 2003). This finding resulted in studies using third party donors without HLA matched without impact in clinical outcome (Le Blanc et al., 2008; Kebriaei et al., 2009). Third party donor cells have the advantage of prior cryopreservation, allowing cell product availability in just few days after a clinical indication of MSCs treatment.

Most GVHD studies use bone marrow aspirate as a source of MSCs production. Of 30 clinical studies assessed in the literature, 25 used bone marrow MSCs (18 of which for GVHD treatment and seven for GVHD prevention). Four studies using umbilical cord-derived MSC to prevent GVHD and one study using adipose-derived MSCs to treat GVHD have also been reported. These studies have compared MSCs treatment only with conventional treatment or historical control. Thus far, no studies have compared the impact of different biological sources of MSC in clinical outcome. Variabilities in trial design is one main limitation for carrying out a meta-analysis to determine which biological source is better or if they are all equivalent in terms of adverse effects and therapeutic benefits (Rizk et al., 2016).

## Safety of Msc

Although malignant transformation of MSCs is a theoretical risk, a systematic review conducted by a Canadian group in 2012 found no significant association between MSC infusion and tumor formation. Malignancy were detected only in patients with ongoing malignancies or as recurrence events. No *de novo* malignancies have been reported, although the clinical follow-up in the examined trials were rather short, ranging from 3 to 60 months (Lalu et al., 2012).

Another meta-analysis of clinical trials enrolled seven studies with a total of 593 patients (334 treated with MSCs and 259 without MSC treatment). Infusion was safe and well tolerated in all studies (doses ranging from 0.1 to 10 million cells per kg) and there was no report of oncogenesis in the follow-up period. Trials using MSCs prophylactically had a median follow-up of 10–60 months. Follow-up of trials using MSCs as treatment varied from 2 to 23 months (Fisher et al., 2019).

As immunosuppressive treatment can reduce graft-versus-leukemia (GVL) effect, two studies reported relapse of malignant disease in short follow up (Kebriaei et al., 2009; Martin et al., 2010). However, there is insufficient evidence to determine this association with high risk of malignant relapse (RR 0.83, 95%, CI 0.37–1.84; participants 275; studies 2) (Fisher et al., 2019). Also in long term follow up, six trials reported this complication but there is insufficient evidence to associate this risk with MSC infusion (RR 1.08, 95%, CI 0.73–1.59; participants 323; studies 6).

Safety regarding the use of MSCs also involves evaluation of possible complications related to embolism in capillary-rich organs. Among the most common routes of MSCs delivery, including topical application, intramuscular or direct injection into organs, intravenous and intra-arterial infusion, the preferred route is the intravenous (Moll et al., 2019). Despite its simplicity, possible complications are related to embolism or thrombus formation. Indeed, Wu et al. (2017) reported that two patients with renal transplantation and chronic kidney disease presented thromboembolism after infusion of MSCs derived from umbilical cord.

A study by Cui et al. (2015) showed in a mouse model that cerebral blood flow was reduced when infusing high amounts of MSCs, which could lead to more severe embolism events. They also showed that not only the number of MSCs is important for this effect but also the speed of cell infusion. The authors demonstrated that longer infusion times were more related to embolism complications. However, given that the follow-up period of this study was limited to 3 days, the authors suggest that these complications could be a transient event.

In another experimental model, Gleeson et al. (2015) showed that, when infused in female pigs, bone marrow MSCs expressed active tissue factor, a key factor of the soluble coagulation cascade that supports thrombin generation and thrombus formation. To counteract this effect, the authors suggest the use of an antithrombotic therapy when MSCs are administered. Christy et al. (2017) also reported a procoagulant activity of MSCs; they showed that adipose-derived MSCs displayed a higher procoagulant activity than MSCs derived from bone marrow. The authors suggest that MSCs should be tested for coagulation activity and that patients should be monitored for these possible complications.

Altogether these studies indicate that patients in cell therapy protocols should be monitored for possible embolism or thrombus events related to MSCs infusion and, when applicable, antithrombotic therapies could be applied. Preclinical studies like these are of utmost importance to prevent translational failure. To this end, critical parameters such as the therapeutic window, delivery route, type of cells, immunogenicity, comorbidity, and concomitant treatment, should be considered in these studies.

## Efficacy of Msc

Evidence of efficacy is difficult to determine since the clinical studies published so far are highly heterogeneous. Some studies use MSCs as prophylactic scheme, with infusion in pre-determined days. Six studies analyzed incidence of acute GVHD and other six studies evaluated chronic GVHD (Tables 1, 2). Possible therapeutic benefit from MSC treatment seems to occur in chronic GVHD patients, although the quality of evidence is low as studies had different schemes of infusion (Fisher et al., 2019).

**TABLE 1:** Prophylactic trials for acute GVHD.

**Study**	**MSC**		**No MSC**		**Risk ratio**
	**aGVHD events**	**Total participants**	**aGVHD events**	**Total participants**	
Ghavamzadeh et al., 2010	6	25	4	23	1.38 (0.45–4.28)
Kuzmina et al., 2012	4	39	8	38	0.49 (0.16–1.48)
Liu et al., 2011	16	27	16	28	1.04 (0.66–1.62)
Mareika et al., 2016	1	10	3	12	0.40 (0.05–3.27)
Ning et al., 2008	5	10	11	15	0.68 (0.34–1.36)
Wu et al., 2013	4	8	8	12	0.75 (0.34–1.67)

*Adapted from Fisher et al. (2019).*

**TABLE 2:** Prophylactic trials for chronic GVHD.

**Study**	**MSC**		**No MSC**		**Risk ratio**
	**cGVHD events**	**Total participants**	**cGVH events**	**Total participants**	
Gao et al., 2016	17	62	30	62	0.57 (0.35–0.92)
Kuzmina et al., 2012	5	19	6	18	0.79 (0.29–2.14)
Liu et al., 2011	13	27	19	28	0.71 (0.44–1.13)
Mareika et al., 2016	1	10	4	12	0.30 (0.04–2.27)
Ning et al., 2008	4	10	5	15	1.20 (0.42–3.41)
Wu et al., 2013	1	8	5	12	0.30 (0.04–2.11)

*Adapted from Fisher et al. (2019).*

Only two controlled trials evaluated efficacy in acute GVHD treatment without difference in clinical manifestation in both groups (MSCs versus no MSCs). In one study with pediatric patients, complete and partial responses were reported in 58 and 17% patients, respectively (RR 2.0, 95%, CI 0.20–19.6 participants 28). One trial evaluated MSC for treatment of chronic GVHD in 40 patients. In this study, complete and partial responses were observed in 15 and one patients, respectively (RR 5.0, 95%, CI 0.75–33.21) (Fisher et al., 2019).

As for safety, the cell administration route is a variable potentially impacting therapeutic effects. MSC systemic delivery is one of the most common administration route in cell therapy since it does not require an invasive procedure but, on the other hand, it relies on a transendothelial cell migration process toward the lesion site, which could have a direct influence in the treatment efficacy Nitzsche et al. (2017). MSCs take a longer time to complete diapedesis than leukocytes (Teo et al., 2012) and this process is not improved by increased persistence of circulating MSC, affecting the amount of cells reaching the targeted organs (Schmidt et al., 2006).

Mesenchymal stromal cells should be able to exit circulation and migrate to tissues/organs in order to repair local lesions due to GvHD. This process initially involves a contact between the cells and the endothelium and, subsequently, transmigration toward the target tissue/organ. MSCs rolling depends on presence of platelets (Teo et al., 2015) and different ligands such as P-selectin (Rüster et al., 2006), glycoproteins and galectin-1 (Suila et al., 2014). MSCs also express a variety of integrins, which can be responsible for the rolling process (Nitzsche et al., 2017). After this first step, MSCs should adhere firmly to the endothelium, which is supported by the expression of various chemokines such as CCR2, CCR4, CCR7, CCR10, CXCR5, CXCR6, and CXCR4 (Andreas et al., 2014); immediately after adhesion, MSCs reorganize the cytoskeleton inducing a polarization before transmigration (Belema-Bedada et al., 2008). MSCs migration to the subendothelial space is mediated by integrins and metalloproteinases that breakdown the basal lamina (Cheng et al., 2012).

Enhancing migration and homing of MSCs to target tissue/organ could be achieved by genetic engineering these cells to increase the expression of chemokines, integrins or selectins (Nowakowski et al., 2013). This approach could help increasing the effectiveness of MSC in GvHD patients.

## Dose of Msc

One of the earliest trials of MSC infusions in humans occurred in 1995 (Lazarus et al., 1995), in which patients with hematologic malignancies and in complete remission received a one-time infusion of autologous bone marrow MSC at doses of 1, 5, or 50 × 10^6^ cells. Since this pioneer study, many others have been performed over the last decade establishing an excellent safety profile for both, autologous and allogeneic MSC infusions, over a range of cell doses and different schemes (Table 3).

**TABLE 3:** Dosing scheme of selected clinical studies testing MSCs for treatment of GVHD.

**References**	**Acute or chronic**	**Source of MSCs**	**Dose**	**Scheme**
von Bonin et al., 2009	aGVHD	Allogeneic	0.9 × 10^6^/kg	Weekly
Jitschin et al., 2013	aGVHD	Allogeneic	2 × 10^6^/kg	Single dose
Shipounova et al., 2014	aGVHD	Allogeneic	1.2 × 10^6^/kg	Single dose
Maziarz et al., 2015	aGVHD	Allogeneic	1–10 × 10^6^/kg	Single dose or weekly
Kebriaei et al., 2009	aGVHD	Allogeneic	2 or 8 × 10^6^/kg	Two infusions
Gao et al., 2016	cGVDH	Allogeneic	3 × 10^7^/kg	Monthly (four times)
Kuçi et al., 2016	aGVHD	Allogeneic	50–129 × 10^6^	Not specified
Yin et al., 2014	aGVHD	Allogeneic	2 × 10^6^/kg	Weekly (three times)
Bader et al., 2018	aGVHD	Allogeneic	1–2 × 10^6^/kg	Weekly (one to four times)
Salmenniemi et al., 2017	aGVHD or cGVHD	Allogeneic	2 × 10^6^/kg	Once or biweekly (six times)
Dotoli et al., 2017	aGVHD	Allogeneic	2 × 10^6^/kg	Weekly (three times)
von Dalowski et al., 2016	aGVHD	Allogeneic	0.99 × 10^6^/kg	Two times
Kurtzberg et al., 2014	aGVHD	Allogeneic	2 × 10e6/kg	Biweekly (eight times)

For most clinical indications, human MSCs are transfused intravenously at doses typically in the one to two million cells per kilogram. For instance, Le Blanc et al. (2008) treated 55 acute GVHD patients with a median dose of 1.4 × 10^6^ cells per kg. Almost a half of them received two doses and six patients, three to five doses. Complete responders had lower transplantation-related mortality 1 year after infusion than patients with partial or no response, as well as higher overall survival 2 years after hemopoietic stem cell transplantation (Le Blanc et al., 2008).

The Osiris trial Protocol 260 compared two different doses of MSCs for acute GVHD. Patients were randomized in two arms: high-dose (8 × 10^6^ MSC/kg) or low dose (2 × 10^6^ MSC/kg). Patients were stratified for dose levels between grades II and grades III/IV of acute GVHD. Standard steroid therapy using glucocorticosteroids and cyclosporine or tacrolimus, was continued at therapeutic dose levels. Seventy seven percent of patients had complete response and 16% partial response (RR 0.76 CI 0.51–1.14 for complete response, RR 11.69 CI 0.7–194.79) without difference in both arms (Kebriaei et al., 2009; Fisher et al., 2019).

For chronic GVHD patients, a team from Karolinska University Hospital, Stockholm, Sweden, reported a study with 11 patients who received six doses of 2 × 10^6^ MSC/kg, at a 4–6-weeks interval. Patients who responded and tolerated the initial six dose regimen received one to three additional MSC doses. Two patients have discontinued all systemic immunosuppression and another two patients were free of steroids and tapering calcineurin inhibitors. The median follow-up time of this study was 29 months. Quality of life was evaluated using the FACT-BMT questionnaire, and responders showed a mean increase in FACT-BMT total score of 6.6 points, or 8%, compared to baseline values, at last follow-up (Von Bahr et al., 2015).

A recent Cochrane review identified 12 studies and 13 ongoing trials involving adult or pediatric patients with GvHD (acute or cronic). In these studies, patients were treated with MSCs doses ranging from 10^5^ to 10^7^ cells/kg, but no differences in clinical outcome could be associated with MSC dose (Fisher et al., 2019).

## Production of Mscs Under Gmp Conditions for Clinical Use

**FIGURE 3:**
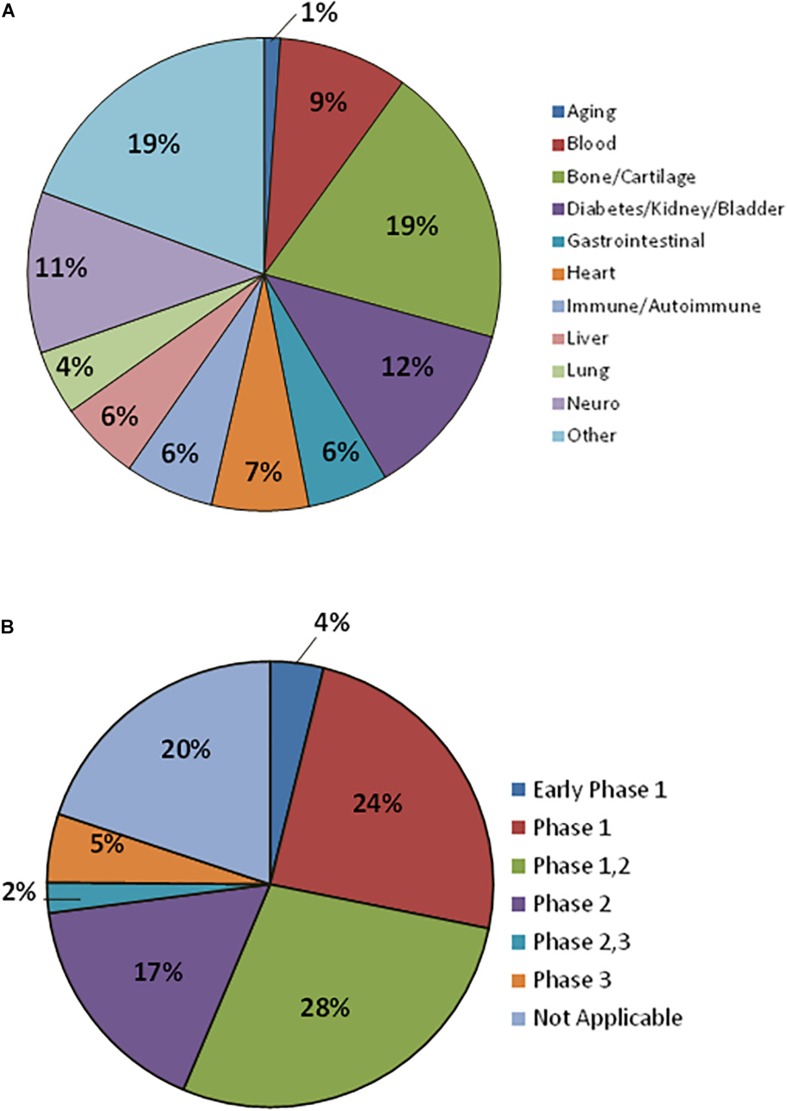
Mesenchymal stromal cells in Clinical Trials. **(A)** Therapeutic indications being addressed with MSCs. **(B)** MSCs Clinical Trials classified by Clinical Phase. Data for 181 registered clinical trials with recruiting status.

The MSCs anti-inflammatory properties as well as homing to sites of inflammation, immunomodulatory properties, and trophic influence on tissue repair, have made these cells very popular for clinical studies (Trounson and McDonald, 2015; de Witte et al., 2018). Up to February 2019, there were 936 registered clinical trials using MSCs with 181 recruiting status (Figure 3). Most of these MSCs clinical trials use allogeneic cells and these trials have the highest activity in United States, Europe, and China. Conditions more frequently indicated for MSC therapy include degenerative osteoarthritis, defect of articular cartilage, rheumatoid arthritis, GVHD, sickle cell disease, thalassemia, leukemia, nephrotic syndrome, liver cirrhosis, diabetes mellitus, lupus, Crohn’s Disease, multiple sclerosis, amyotrophic lateral sclerosis, autism spectrum disorder, ischemic heart disease, among many others. Although there are a high number of ongoing clinical studies, only few MSC commercial products are approved by regulatory agencies (Table 4).

**TABLE 4:** Commercially approved MSCs-based products.

**Medicinal product**	**Company**	**hMSC type**	**Clinical indication**	**Marketing authorization**
Allostem	AlloSource	Allogeneic hASC	Bone regeneration	FDA (United States)
Cartistem	Medipost	Allogeneic UCB-MSC	Traumatic and degenerative osteoarthritis	SKFDA (Korea)
HeartiCellgram	FCB-Pharmicell	Autologous BM-MSC	Acute myocardial infarction	SKFDA (Korea)
Grafix	Osiris Therapeutics	Allogeneic BM-MSC	Soft tissue defects (acute and chronic wounds)	FDA (United States)
Prochymal	Mesoblast	Allogeneic BM-MSC	GVHD	Health Canada (Canada); Medsafe (New Zealand)
OsteoCel	NuVasive	Allogeneic BM-MSC	Spinal bone regeneration	FDA (United States)
OvationOD	Osiris Therapeutics	Allogeneic BM-MSC	Bone regeneration	FDA (United States)
Cartiform	Osiris Therapeutics	Allogeneic BM-MSC	Cartilage repair	FDA (United States)
Stravix	Osiris Therapeutics	Allogeneic BM-MSC	Wound repair	FDA (United States)
Cupistem	Anterogen	Autologous hASC	Crohn’s fistula, reduce inflammation and regenerate damaged joint tissues	SKFDA (Korea)
TEMCELL	Mesoblast	Allogeneic BM-MSC	Acute radiation injury, Crohn’s disease, GVHD, type I diabetes, and myocardial infarction	PMDA (Japan); Health Canada (Canada); Medsafe (New Zealand)
Trinity evolution	Orthofix	Allogeneic BM-MSC	Bone regeneration	FDA (United States)
Trinity elite	Orthofix	Allogeneic BM-MSC	Bone regeneration	FDA (United States)
QueenCell	Anterogen	Autologous hASC	Regeneration of subcutaneous adipose tissue	SKFDA (Korea)
Ossron	RMS	Autologous BM-MSC	Bone regeneration	SKFDA (Korea)
Alofisel	TiGenix/Takeda	Allogeneic hASC	Complex perianal fistulas	EMA (Denmark)

Currently, distinct strategies are used to produce human MSCs *ex vivo* for clinical purposes, as an alternative method for regenerative therapy in many diseases. In any case, regulatory issues related to the safety, efficacy and quality of MSC therapies must be considered while preparing a cell- or tissue-based product for clinical and commercial use. Quality assurance (QA) and quality control (QC) must be provided in any cell manipulation under good manufacturing practices (GMP) grade.

Thus far, there is no consensus on the production of MSCs regarding GMP system. The QC standards may be discussed individually on each project following the local regulatory agency authority. In Brazil, the regulatory agency (ANVISA – Agência Nacional de Vigilância Sanitária), establish specific criteria for advanced therapy products to assure the quality, safety and efficacy of cell therapy in the country (Resolutions: RDC 214/2018 and RDC 260/2018). Cell viability, cytogenetics, potency tests and sterility tests to verify contamination by mycoplasma, aerobic and anaerobic bacteria, fungus, and special microorganisms such as filamentous fungus, are routinely used to ensure the quality of the cell product. Main GMP issues addressed by regulatory agencies in different countries are comparable and can be assessed in Supplementary Table S1.

According to the Foundation for the Accreditation of Cell Therapy (FACT) and Brazil’s ANVISA, cytogenetic testing is mandatory to release a cell product. Most manufacturing units perform karyotyping on the final cell product and the results are an important component of the release criteria. In case of any chromosomal abnormalities in the manipulated cells, the incoming samples may be karyotyped to ensure that the donor does not have any constitutive chromosomal abnormalities and that the alteration has probably originated in a cell clone during *ex vivo* expansion. A study by Nikitina and colleagues estimated that around 10% of MSC samples contain chromosomal aberrations after expansion, although the clinical consequences of such aberrations are unknown (Nikitina et al., 2018).

However, since the potential clinical impact of minor changes in karyotyping is difficult to evaluate, not all services consider the cytogenetic testing as a release criterion (Philippe et al., 2010; Tarte et al., 2010; Hanley et al., 2013).

There are many different variations in existing manufacturing protocols for MSC production that may influence the final characteristics of the cells. The type of media supplementation is a typical example. Fetal bovine serum (FBS) is the most common supplementation used. FBS concentration in media ranges from 2 to 20%, with 10% FBS being the most common concentration. This variability on FBS concentration may result in different amounts of growth factors to stimulate cell survival and proliferation (Carmen et al., 2012; Mendicino et al., 2014). Xeno-free media supplementation is also used, including human platelet lysate and human serum. Some cell culture media are fully defined and do not require extra supplementation. However, the lack of serum or platelet lysate could impair MSCs attachment to the surface and there is a need of a coating substrate that is derived from animal or human tissue. In our GMP unit, we have validated the use autologous serum (patient-specific) to expand MSC products for infusion in patients enrolled in official clinical protocols. Under such condition, typically, a 20 mL bone marrow aspirate yields 10 million MSCs after three cell culture passages.

Many efforts have been made to develop technologies to achieve production of adequate number of cells with high therapeutic quality. *Ex vivo* MSC expansion may be performed by conventional cell culture techniques or by using bioreactors. Considering autologous use, it is possible to produce lower cell quantity and the scale-out approach can be performed, using planar culture systems with multiple flasks in cell factories. For allogeneic use, MSCs can be expanded to a large number of cells in bioreactor systems (scale-up approach) and cryopreserved in cell banks for future use (Pittenger et al., 1999; dos Santos et al., 2013; Mizukami and Swiech, 2018). However, it is critical to determine cell viability, potency and sterility post-thawing to validate the cryopreservation method before routine implementation (Galipeau, 2013; Mendicino et al., 2014).

Monolayer culture is the traditional technique for MSC expansion; however, excessive manipulation may interfere in the functional properties of cells due to enzymatic treatments in many passages, higher contamination risk due to intense manipulation, prolonged culture to generate adequate amounts of cells, impairing cell physiology. Scale-up based cell expansion meets the criteria for GMP with quality standards, allowing monitoring of pH, temperature, carbon dioxide, and oxygen concentration over time, and maintenance of cells behavior (adherent or suspension cells). There are several bioreactors available for cell expansion such as stirred tank bioreactor, rocking bioreactor, hollow fiber bioreactor, and fixed bed bioreactor. The choice of bioreactor will depend on the aim to be achieved, i.e., final amount of cells required for infusion, type of cell (adherent or suspension growing cells), mid/long term use, etc (Jung et al., 2012; Clarke, 2013; Mizukami and Swiech, 2018).

After MSC expansion, cells are harvested by using an enzyme, usually trypsin. Since this enzyme is normally of porcine origin, alternative GMP recombinant enzymes are available and their use should be prioritized. Mechanical detachment of cells cultured under GMP facilities is also possible by using a cell scraper tool, although detachment must be done gently in order to avoid cell damage and death. While enzymatic MSC detachment can be adapted in expansion protocols using bioreactors, the same adaptation is harder to achieve for mechanical detachment techniques.

When high MSC doses and multiple cell infusions are required, which is typically the case of GVHD treatment, cryopreservation of previously expanded cells is an optimal solution to have high amounts of GMP-grade cells readily available for infusion. Nonetheless, cryopreservation is a critical step in MSC manipulation. Although either cryopreservation bags or regular cryotubes could be used for this purpose, the use of the latter is limited by the low volume of cell preparation that can be stored per vial (usually 1.5–2 mL). Cryopreservation media usually contain DMSO as cryoprotectant but it can cause some adverse effects when infused in patients. Typically, cells are stored using a ratio of 10% DMSO and 90% of serum. Thus, after thawing, some laboratories centrifuge and wash cells to remove DMSO. An alternative is to use cryoprotectants containing methylcellulose, sucrose, trehalose, glycerol, hydroxyethyl starch, and polyvinylpyrrolidone. Some companies already developed serum-free and xeno-free cryopreservation media (e.g., Cryostor – StemCell Technologies, Plasmalyte-A – Baxter and Synth-a-Freeze – Gibco) to circumvent toxicity. Importantly, validation of progressive freezing and thawing cycles is required to avoid significant loss of cell viability.

Despite the lack of consensus in critical steps of MSCs production under GMP conditions, different protocols are available to attain high yields of expanded MSCs. However, these *ex vivo* expanded MSC need to meet the quality standards required by regulatory agencies.

## Informative Mscs Potency Assays for Use in Gvhd Treatment

Cellular products intended for clinical use must also pass functional evaluation, a key part of a GMP quality control program. Currently, there is no specific release criterion required by the authorities for testing MSC potency. The most used and accepted potency tests for MSCs evaluate their ability to differentiate into three different cell types (osteoblasts, adipocytes, and chondroblasts), to inhibit T-lymphocyte proliferation or to promote endothelial tube formation.

All these tests involve *in vitro* assays that can be easily adapted for routine screening of cell preparations. Nonetheless, adoption of different assays for extensive functional characterization of cells is expensive and time consuming, which may delay the availability of freshly produced MSCs for infusion in patients at critical clinical conditions. Alternatively, a key test could be performed to evaluate a specific MSC property that is correlated with the aimed therapeutic effect. To this end, knowledge of the mechanism of cell action *in vivo* is of fundamental importance, which reinforces the relevance of basic stem cell biology studies.

For instance, exploration of MSC immunosuppressive potential is well established in immunological-based diseases such as diabetes, multiple sclerosis, and GVHD. For this type of application, assaying MSC immunomodulatory activity would better suit a potency test then assaying MSC multipotency. This could be achieved by co-culture assays with T cells to evaluate MSC effects on T cell proliferation and/or by cytokine release by MSC (Wuchter et al., 2015; Mizukami and Swiech, 2018).

Di Nicola et al. (2002) showed that MSCs were able to suppress T-cell proliferation when these cells were added to mixed lymphocyte reactions. They also demonstrated that MSCs were able to inhibit both CD4^+^ and CD8^+^ cells. This study suggested that cell-cell contact between MSC and effector cells was not necessary to inhibit T-lymphocyte proliferation (Di Nicola et al., 2002). However, cell-cell contact was important for T-cell arrest in G0 phase of the cell cycle (Glennie et al., 2005). MSC do not seem to induce T-cell apoptosis *in vitro* (Krampera et al., 2006).

The immuno-suppressive activity of MSCs may also be determined after exposure to IFN-gamma. In this assay, presence of primed MSC could be indicated by expression of MHC class I, MHC II, PDL-1, or other modulatory molecules. Release of certain chemokine receptors such as CXCR3, CXCR4, CXCR5, and CCR7 could also be assayed to indicate immunomodulatory active MSC (Krampera et al., 2013). As previously addressed, potency tests based on the cytotoxicity of recipient’s cells toward donor MSCs is another example of informative test associated with clinical response of GVHD patients (Galleu et al., 2017). Incorporation of such assays in the routine QA tests is feasible, since it would only require basic flow cytometry and ELISA platforms, or similar alternatives.

Specific MSC properties involved in their expected therapeutic effect may be affected by different factors, including cryopreservation (Weise et al., 2014). Thus, proper potency assays are also valuable to assess stability of cell therapy products at different storage conditions over time. Donor age is another factor that influences MSC activity. Stolzing et al. (2008) analyzed expression of cell surface markers, oxidative cell damage and senescence in MSCs derived from adults and children. They have found a reduction in CFU-F (colony forming unit-fibroblast) generation and in the subset of CD45^low^/D7fib^+ve^/LNGF^+ve^ cells in samples derived from adults compared with samples derived from children. MSCs obtained from elderly people also showed increased levels of ROS, p21, and p53 proteins. The authors suggested that active MSCs derived from bone marrow decrease in number with age and that these cells are not as potent as the ones isolated from younger patients. Such aging effect on MSC properties should be considered when defining inclusion criteria for MSC donors in cell therapy protocols.

## Concluding Remarks

Evidences in the literature support safety of MSCs treatment for GVHD, whereas efficacy of this type of cell therapy still needs further clarification. Efficacy of MSC-based treatment is more evident for chronic GVHD patients. Double-blind randomized controlled trials with steroid-refractory GVHD patients should be designed to better address MSCs efficacy. Well-defined inclusion/exclusion criteria for patient accrual and a standardized protocol for GMP production of MSCs should facilitate multicenter studies and acquisition of faster results for clinical outcome assessment. Regarding MSCs production, uniformity in donor age and automated *ex vivo* expansion of cells should help minimize product variability. Also, the choice of potency test is critical in evaluating suitability of the final product, since the desirable therapeutic effect may differ among clinical trials. In the case of GVHD, potency tests addressing MSCs immunomodulatory activity are key factors for obtaining high quality MSCs for therapeutic purposes. Allogeneic MSCs transplantation seem highly appropriate for GVHD treatment, due to the poor clinical condition of patients for tissue donation and lack of necessity of HLA match. In this scenario, the clinical use of cryopreserved third party MSC products offers the additional advantage of faster cell product availability, compared with autologous transplantation. Additional tests to address the cytotoxicity of recipient’s cells toward allogeneic MSCs should help refining the selection of eligible patients. Distribution of MSC products stored in cryobanks to different hospitals is also feasible, allowing potential therapeutic benefits for a greater number of patients in need.

## Author Contributions

All authors wrote the manuscript and revised the final submitted version.

## Conflict of Interest

OO was a visiting scholar at Departamento de Hemoterapia e Terapia Celular, Hospital Israelita Albert Einstein.

The remaining authors declare that the research was conducted in the absence of any commercial or financial relationships that could be construed as a potential conflict of interest.
